# Enhanced Near-Field Chirality in Periodic Arrays of Si Nanowires for Chiral Sensing

**DOI:** 10.3390/molecules24050853

**Published:** 2019-02-28

**Authors:** Emilija Petronijevic, Concita Sibilia

**Affiliations:** Department S.B.A.I., Sapienza Università di Roma, Via A. Scarpa 14, 00161 Rome, Italy; concita.sibilia@uniroma1.it

**Keywords:** semiconductor nanowires, chirality, enantioselectivity, near-field optical chirality

## Abstract

Nanomaterials can be specially designed to enhance optical chirality and their interaction with chiral molecules can lead to enhanced enantioselectivity. Here we propose periodic arrays of Si nanowires for the generation of enhanced near-field chirality. Such structures confine the incident electromagnetic field into specific resonant modes, which leads to an increase in local optical chirality. We investigate and optimize near-field chirality with respect to the geometric parameters and excitation scheme. Specially, we propose a simple experiment for the enhanced enantioselectivity, and optimize the average chirality depending on the possible position of the chiral molecule. We believe that such a simple achiral nanowire approach can be functionalized to give enhanced chirality in the spectral range of interest and thus lead to better discrimination of enantiomers.

## 1. Introduction

Chirality, a lack of mirror symmetry [1], is an important property of our world governing the behavior of many molecules, enzymes, DNA, and sugars. Optical isomers of the opposite handedness that are non-superimposable images of each other are called *enantiomers*. Specifically, two enantiomers of the same chiral drug have the same chemical structure and physical properties, but different spatial arrangement and optical activity [2], which leads to differences in biological activities such as toxicity [3,4] and enantioselective reactions [5]. Two enantiomers can therefore have extremely different effects on the human body where one can lead to serious side effects [6,7,8]. Conventionally, polarimetry and circular dichroism (CD) measurements are used to distinguish enantiomers since they differently interact with the circularly polarized (CP) light of the opposite handedness [9]. However, such experiments require high concentrations of enantiomers and long integration times as the intrinsic CD of the molecules is usually extremely low. Therefore, finding novel approaches to detect and recognize low concentrations of enantiomers, thus increasing the sensitivity and reducing the material waste, is of great interest for today’s pharmaceutical industry.

In the past decade, the nanoscale photonics community has been dealing with chirality as well: artificial plasmonic nanostructures with broken symmetry were shown to provide a chiral response and CD [10,11,12,13,14,15,16]. We recently showed that semiconductor-based nanomaterials, asymmetrically covered by thin metallic layers, can also produce strong CD [17,18,19]. Merging of the chiral plasmonics with the field of chiral biomolecule enantioselectivity [20] has already given promising results in chiral sensing. For example, in [21], the authors reported on the several orders of magnitude enhancement of the enantiomer detection by means of twisted plasmonic unit cells periodically arranged to form a chiral *metamaterial*. Furthermore, investigation of the near-field effects has opened ways to enhance the interaction between the nanostructure and the chiral molecule via so called *superchiral* fields [22,23]. Namely, for a monochromatic electromagnetic field at frequency ω the optical chirality factor C is defined as:

(1) C=−0.5ε0ωIm{E*→(r→)·B→(r→)},

where $\vec{E}$ and ($\vec{B}$) are electric (magnetic) complex field amplitudes. For a plane wave, C is zero for linear polarization, maximum for right circular polarization (RCP), and minimum for left circular polarization (LCP), switching the sign between RCP and LCP. In the near-field of nanostructured materials at the resonance, the electric and magnetic fields can be parallel and out of phase, while their enhancement leads to a C greater than the one of CP, hence the term superchirality. The C factor directly influences the excitation rate of chiral molecules [24] as well as the absorption dissymmetry [25], and its switching of the sign between RCP and LCP is directly connected with the enantioselectivity. Namely, the difference in the absorption rate of two enantiomers can be significantly enhanced if the medium where the mixture exists has an enhanced C. If the medium is excited with CP so that it generates enhanced C of one sign, this will enhance the absorption rate of one enantiomer. Changing the handedness of CP will enhance the absorption rate of another enantiomer, leading to the enhanced difference between the two enantiomers. Finally, in this way the sensitivity of distinction for low enantiomer concentrations can be improved. In general, the medium should produce enhanced C in the volume of the enantiomers, and it must be of the same sign over the volume in order to contribute to the absorption rate of only one enantiomer. Plasmonic nanostructures can be designed to tailor C in the wavelength region of interest for a particular chiral molecule. We recently numerically investigated C enhancement in GaAs nanowires (NWs) asymmetrically covered by Au [26], while in [25,27,28], symmetric nanostructures were shown to give an enhanced near-field C as well. Moreover, in [25] the authors underlined the importance of having only near-field, local chiral effects, without the background chirality of the supporting medium itself. In this way, all the chiral enhancement and corresponding effects arise from the coupling of the chiral molecule with the electromagnetic field in the vicinity of the achiral structure.

In this work, we investigate intrinsically achiral 2D periodic arrays of Si NWs on a Si substrate, a device which is completely symmetric and easy to fabricate. Geometric parameters can be chosen so that these NWs support leaky waveguide modes [29,30,31] in the visible part of the spectrum. These modes are weakly guided along the NW borders and have a strong field leakage to the surrounding medium, where the evanescent field components can produce a near-field chirality. In [26] we investigated GaAs NW modes at ~800 nm, where we showed that for linearly polarized excitation, the near-field chirality cancels out and the additional symmetry breaking (e.g., the asymmetric layer of Au) must be introduced in order to have a C of prevalently one sign. In [32], NW dimers are used to induce the optical chirality and the hotspots of high C enhancement. Here, we show that under circularly polarized (CP) excitation, achiral NW structure can generate the enhanced chirality in the NW border vicinity, without the additional symmetry breaking which would complicate the processing of a perspective device. For Si NWs, we investigate the influence of geometric parameters on the mode wavelength, which in turn tailors the C distribution; we show that it is possible to spectrally place the enhanced C in the high frequency range of the visible spectrum, technologically relevant for chiral molecules. Further, we investigate the optimization of the average C so that it is distributed in the big part of the volume of the unit cell. For the opposite handedness of the CP excitation, average C changes sign, thus we propose these metasurfaces for enantioselectivity applications, with parameters that can be fixed in the wavelength range of interest. As these materials are easy to fabricate and implement in the existing technology, we believe that this approach can lead to efficient background-free tunable chiral recognition, with decreased waste of materials and increased efficiency.

## 2. Results

**Figure 1:**
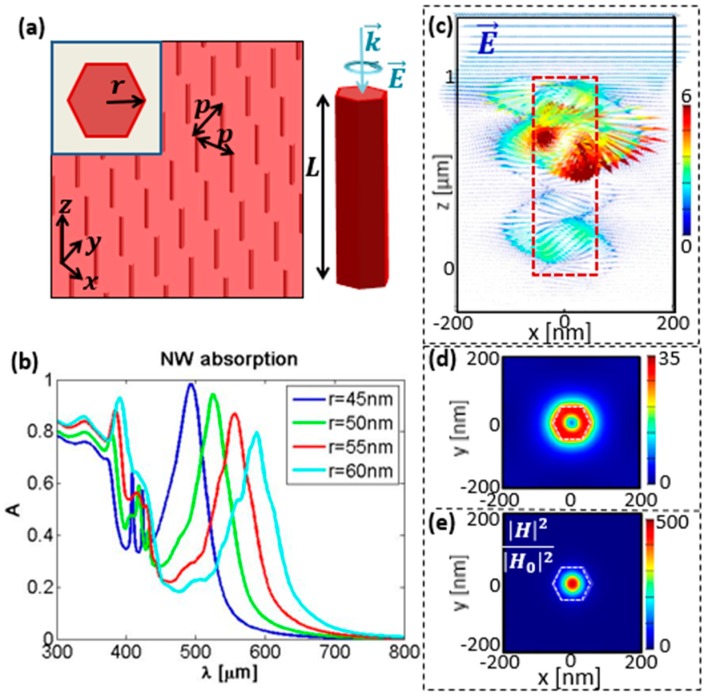
(**a**) Schematics of the nanowire NW array with quadratic unit cell of periodicity p *x* and *y* directions, and a single NW with diameter D and length L. (**b**) Absorption of the NW arrays with p = 400 nm, and L = 1000 nm, for D = 45–60 nm, for left circular polarization (LCP) excitation. (**c**–**e**) LCP excitation for r = 50 nm at the resonant wavelength λ~525 nm: (**c**) *xz* cross-section of the distribution of the electric field vector; *xy* cross-section of the unit cell at z = 800 nm; (**d**) electric field intensity enhancement; (**e**) magnetic field intensity enhancement.

Structures under investigation are hexagonal Si NWs, that can be patterned on Si substrate, e.g., by means of conventional electron beam lithography [32,33]. In what follows, we use a commercial-grade simulator based on the 3D Finite Difference Time Domain (FDTD) method by Lumerical [34] to simulate complex electromagnetic fields in the Si NW array, and later extract C factor; more details are given in Section 4. The scheme of the simulated device is given in Figure 1a: vertical hexagonal NWs of radius r and length L are periodically arranged with periodicity p in *x* and *y* direction. Such structures have been previously proposed for absorption enhancement, lasing and solar cells, as they can efficiently absorb the visible spectrum wavelengths. The structure is excited by a circularly polarized plane-wave under normal incidence. In Figure 1b we show the absorption spectra dependence on the NW radius, for L = 1000 nm, and p = 400 nm. As expected from theory [35], as well as experiments in [30,31], there is always a fundamental HE_11_ mode excited for these thin NWs, which leads to the resonant absorption; moreover, with the radius increase, the resonant wavelength of the mode red-shifts. For this periodicity, the mode closely resembles the leaky waveguide mode of the single Si NW. In Figure 1c, the electric field vector in *xz* cross-section of the unit cell is shown for the NW array with parameters r = 50 nm, L = 1000 nm, p = 400 nm, excited at its resonance λ = 525 nm with CP light. Clearly, the electric field keeps circularly polarized behavior, while it experiences enhancement, especially in two regions of the volume corresponding to the antinodes of the Fabry–Perot resonances (due to the boundary conditions at the top and the bottom of the NW). In Figure 1d, we show the electric field intensity enhancement at z = 800 nm *xy* cross-section of the unit cell; there is an obvious leakage of the mode to the surrounding medium, along with a 35-fold increase. In Figure 1e, for the same cross-section, the magnetic field enhancement is even higher, but it remains confined in the NW core. According to Equation (1), the enhanced C can be expected at the points where these field enhancements spatially overlap, while $\vec{E}$ and $\vec{H}$ have parallel components out of phase. In the close vicinity of the NW, molecules present in the surrounding medium will experience both electric and magnetic field enhancement, while for the near-field chirality calculations, their phase difference must be considered as well. Thus, in the following, we investigate C distribution dependence on the NW parameters at the resonant wavelengths; we report on the normalized C* = C/C_0_, where C_0_ is extracted for the RCP excitation of the simulation domain without the Si NWs. For better visualization, we show only the points with |C*| > 1.2. 

### 2.1. Influence of the NW Core Radius

**Figure 2:**
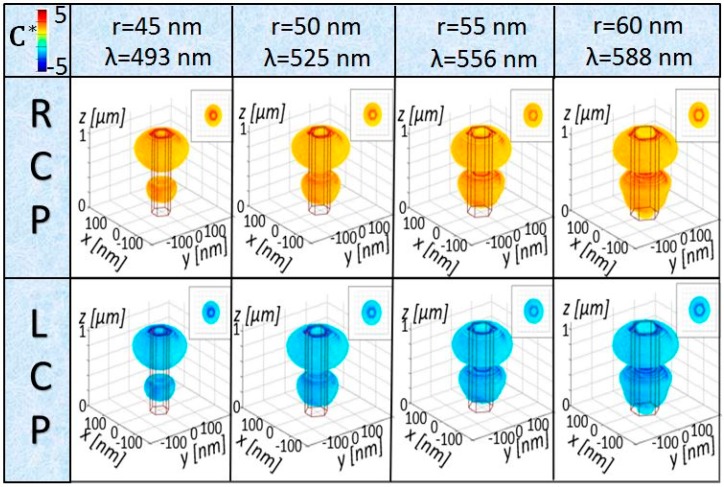
C* distribution in the unit cell for p = 400 nm, L = 1000 nm, and r = 45–60 nm, at the corresponding NW resonant wavelength for RCP (upper, positive) and LCP (bottom, negative) excitation. The insets show the *xy* top view of each C* distribution.

Firstly, C distribution in the unit cell is investigated for p = 400 nm and L = 1000 nm, for the radii investigated previously. In Figure 2, in all the cases, |C*| is higher than 5 at the NW borders and remains enhanced in the NW proximity, where the chiral molecules can be deposited for the experiment. More importantly, for RCP (LCP) excitation, C* keeps the positive (negative) sign of the excitation (normalization by RCP), while having increased values. As the whole domain is achiral, the resonant wavelengths for RCP and LCP are the same. The NW array with r = 45 nm has a resonant wavelength of λ = 493 nm, where Si has higher losses; therefore, the field is efficiently absorbed in the upper part of the NW, and leads to lower electromagnetic field enhancement on the borders closer to the bottom of the NW. For r = 50 nm, we can note that the z positions of the maximum C* correspond to the antinode enhancements in Figure 1c. With the radius increase leading to the resonance red-shift, the losses of the mode become lower, and C* spreads more in the unit cell volume; moreover, the second antinodal enhancement of C* (close to the substrate) also becomes more prominent. Therefore, tuning of the radius can effectively tailor the enhanced C* in terms of wavelength and 3D distribution. As an example, if the enhanced enantioselectivity experiment involved enantiomers with absorption dissymmetry around 590 nm, the last NW with r = 60 nm should be chosen from these four configurations (the enhanced |C*| has the highest spread in the unit cell volume).

### 2.2. Influence of the NW Array Periodicity

**Figure 3:**
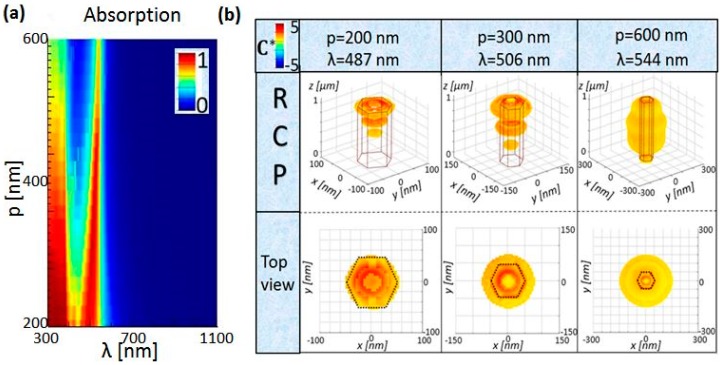
(**a**) Absorption dependence on the NW array periodicity for r = 50 nm and L = 1000 nm. (**b**) Upper part: C* distribution in the unit cell for p = 200 nm, p = 300 nm, and p = 600 nm at the corresponding NW resonant wavelengths for RCP excitation; bottom: *xy* top-view of the corresponding distribution from the upper part. Black hexagonal line shows the geometric position of the NW *xy* cross-section. Only RCP excitation is shown as LCP leads to the sign inversion.

As the coupling between the neighboring NWs influences the electromagnetic fields, we further investigated the absorption and C* dependence on the periodicity (Figure 3a) for r = 50 nm and L = 1000 nm. 2D periodic NW arrays are photonic crystals, where, for larger periods, the single NW modes are not considerably influenced by the array coupling [29,35]. However, for denser Si NW arrays, we note a blue-shift of the resonance with the decreasing period; this arises from the destructive coupling between the neighboring NWs due to enhanced near-field evanescent wave interaction. Unfortunately, in Figure 3b, for p = 200 nm, this leads to C* confinement inside the NW, which is detrimental for the applications where the enantiomers surround the NW in the unit cell. The situation can be optimized by increasing the periodicity, which confines C* also around the NW (e.g., p = 300 nm in the middle of Figure 3b, or p = 400 nm in the second graph in Figure 2). Finally, sparse arrays of p = 600 nm have stronger C* enhancement over the high part of the volume, as it will be shown later. This is due to the strong electromagnetic field enhancement in the near-field medium close to the NW, for the fundamental leaky waveguide modes of the single NW. 

### 2.3. Influence of the NW Length

**Figure 4:**
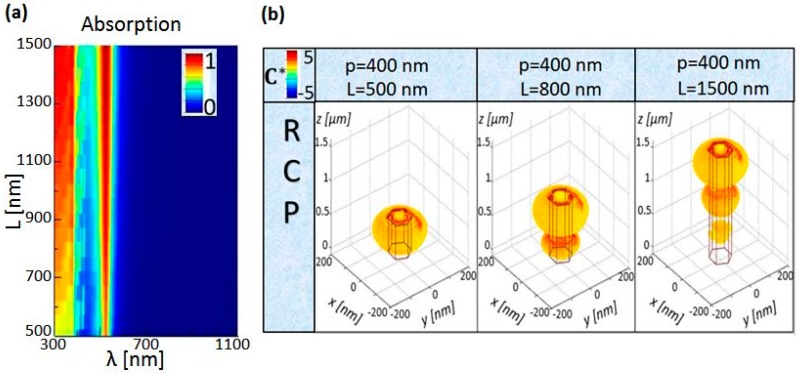
(**a**) Absorption dependence on the NW length for r = 50 nm and p = 400 nm. (**b**) C* distribution in the unit cell for L = 500 nm, L = 800 nm, and L = 1500 nm at the resonant wavelength of 525 nm. Only RCP excitation is shown as LCP leads to the sign inversion.

For the enhanced enantioselectivity, as previously discussed, the nanostructures can be optimized in order to interact with the enantiomers in the most efficient way, which depends also on the enantiomer position. Therefore, we investigated the NW length influence on C* distribution, for r = 50 nm and p = 400 nm. In Figure 4a, the modes do not shift with increasing length, but the unitary absorption is reached only for longer lengths. This is rather expected for vertical high refractive index nanowires, where the absorption peak arises due to the mode, which is mainly defined by the radial boundary conditions of the nanowire core (as in dielectric waveguides). Therefore, the radius plays a major role in the spectral position of the absorption peak, while other geometric parameters have a minor influence, as we experimentally demonstrated in [17,18,30,31]. As expected, the shortest NW (L = 500 nm) has an enhanced C* only due to the first antinode of the resonant mode, while for L = 800 nm another antinode appears. Finally, for L = 1500 nm, three enhanced volume parts are distinguishable; however, the bottom one would not contribute to the enantioselectivity as its C* is more confined inside the NW. If we consider e.g., the enantiomers with absorption dissymmetry around 525 nm, the best Si NW substrate (with r = 50 nm and p = 400 nm) would be the one with L = 500 nm, and the matrix with chiral molecules should be deposited between z = 0 nm and z = 500 nm. Otherwise, if the NW length is fixed to L = 1500 nm, in order to use zones with the highest C*, one should deposit another non-absorbing and achiral medium (buffer layer) in the range 0 nm < z < 1000 nm, and then the chiral substance in the range 1000 nm < z < 1500 nm, which complicates the simple approach proposed here. 

## 3. Discussion

**Figure 5:**
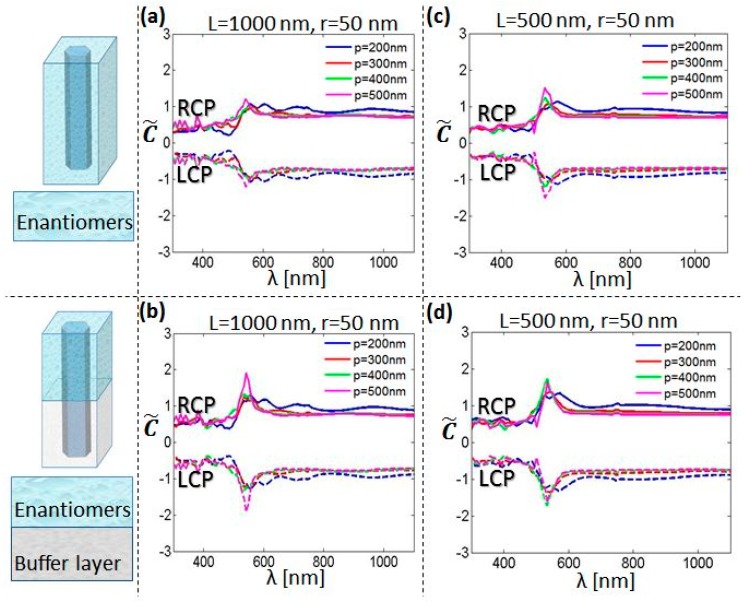
Normalized averaged C spectra for r = 50 nm, p = 200–425 nm, and RCP (full lines) and LCP (dashed lines) excitation. Upper and bottom sketches correspond to the integration from z = 0 nm to z = L, and z = L/2 to z = L, respectively. (**a**) L = 1000 nm, total integration; (**b**) L = 500 nm, total integration; (**c**) L = 1000 nm, half-integration; (**d**) L = 500 nm, half-integration.

The overall enantioselectivity enhancement will depend on the percentage of the chiral molecules that are positioned exactly in the part of the volume with enhanced C*, i.e., in the NW near-field. This is usually not the case for the periodicities that support leaky waveguide modes, so it is important to estimate the average chirality in the unit cell volume, with subtracted NW volume. In order to gain insight into the spectral behavior of the investigated chirality enhancement, we calculate the average normalized optical chirality as:

(2) $$\tilde{C}\left(\lambda \right)=\frac{1}{C_{RCP}\left(\lambda \right)}\frac{∭C\left(x,y,z,\lambda \right)dV}{∭dV},$$

where we integrate C across the unit cell as follows: in the *xy* plane, only the parts of the unit-cell outside of the NW core are considered, while in the *z* direction, we investigate two possibilities. First, we integrate in the 0 < z < L range (upper sketch of Figure 5), i.e., total integration. Then, we take into account only the top C* antinode, integrating in the L/2 < z < L range (bottom sketch of Figure 5), i.e., half-integration, which corresponds to the case when the enantiomers are specially positioned on a buffer layer in order to experience higher C. We approximate that the buffer layer has negligible influence on the C* distribution, which is valid for low concentrations of chiral molecules, deposited in a solvent which subsequently evaporates. However, generally the buffer layer does change the modal confinement, and the NW array must be reoptimized in terms of r, L and p in order to give enhanced C* once the optical properties of the buffer layer are known. In Figure 5a, the total integration of L = 1000 nm and r = 50 nm, for the periodicities from 200 nm to 400 nm gives almost no enhancement at the resonant wavelengths, and it is vaguely present only for p = 500 nm (the lowest NW coupling, $|\tilde{\text{C}}|∼1.2$). However, the half-integration in Figure 5b improves this difference; here, especially for p = 500 nm, $\left|\tilde{\text{C}}\right|$ reaches almost 2, which is a considerable average enhancement for a 0.5 × 0.5 µm^2^ surface. For shorter NWs, L = 500 nm and r = 50 nm, the total integration in Figure 5c gives $\tilde{C}$ improvement with respect to Figure 5a, due to inclusion of the part of the space where one antinode generates enhanced C* (Figure 4b-left). However, this case does not get improved with the half-integration, Figure 4d.

**Figure 6:**
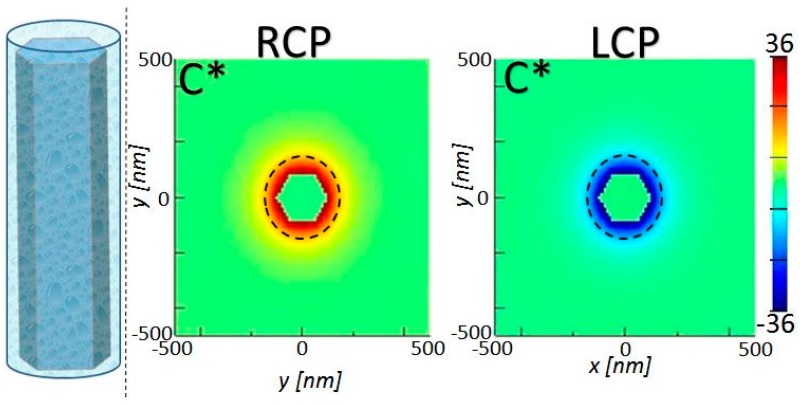
Left: sketch of the Si NW functionalization with bonded chiral substance for the improvement of enantioselectivity. Right: xy cross-section of C* distribution at the antinode of the NW with r = 50 nm, L = 1000 nm, and p = 500 nm (resonant wavelength at 542 nm) for RCP and LCP excitation. Dashed circles represent the volume of the enantiomers attached to the NW surface.

It is worth noting that for all investigated cases in Figure 5, Si NW arrays give a rather modest average optical chirality. However, we underline the advantage of the use of Si in such nanostructures. Apart from the highly developed technology and perfectly known optical and electrical properties, Si NWs have already been proposed for bio and chemical sensing as the NW surface allows for the immobilization of the investigated substance in the NW near-field, thus affecting the sensor performance and enabling high sensitivity and selectivity [36,37]. For the enhanced enantioselectivity, one should position the chiral molecules in a very thin layer close to the surface which enhances the near-field chirality (a few tens of nm, according to [24]). Many approaches have been found to functionalize the Si nanoparticle surface with molecules [38]. Therefore, the ultimate optimization of the presented approach is the attaching of the chiral species to the NW surface, in the small part of the volume as in the sketch of Figure 6. When the solvent evaporates, a small concentration of chiral molecules which remain attached to the Si surface introduces a negligible change in the NW environment, so that the molecules experience the near-field chirality of the resonant modes presented above. Next, we focus on the best case from Figure 5b, i.e., r = 50 nm, L = 1000 nm, and p = 500 nm, which gave the $\left|\tilde{\text{C}}\right|$ peak at 542 nm. In Figure 6 we show the *xy* cross-section of the C* distribution at the resonance and at the z position of the C* antinode, for RCP and LCP excitation (we omit C* inside the NW as it is not important for the interaction with the enantiomers). There is a 36-fold C enhancement at the NW borders, and the chiral molecules in the dashed circles experience the average C at least on the order of 20, which will finally lead to significantly higher enhancement with respect to the approach in Figure 5c. Therefore, one can smartly functionalize Si NW sidewalls so that the enantiomers are positioned at points with maximum C*.

## 4. Materials and Methods 

FDTD simulations solve the Maxwell’s equations over discrete spatial and temporal grid, and give as a result complex electromagnetic fields in the unit cell consisting of one Si NW in the middle. Si NW lie on a Si substrate, which is considered semi-infinite for z < 0; for z > 0, the medium is air. Optical properties of Si were taken from the Lumerical database. In the *z* direction, perfectly matched layers (PML) were placed at least half the maximum wavelength from the top and bottom of the NW to ensure the numerical stability. For the normal incidence excitation, a plane-wave source was used from the top side (negative *z* direction in Figure 1a), and periodic boundary conditions were applied in the *xy* plane. Circular polarization was simulated as a combination of two orthogonal sources with a phase difference of ±90°. Total absorption of the NW was simulated by integrating the absorption per unit volume σ_abs_ over the NW volume; σ_abs_ was calculated from σ_abs_ = −0.5ω^2^|E|^2^Im{ε}, from the electric field and complex refractive index monitors across the NW domain. Electric and magnetic field confinements were monitored by a cross-section field profile monitor in the *xz* and *xy* planes. C factor was extracted from a 3D field profile monitor encompassing the whole unit cell in the *xy* plane, and having a z span from 0 nm to the NW length. The calculated near-field chiral properties arise from electromagnetic field confinement of the NW modes, and are not an intrinsic property of molecules. Moreover, approximation of air as a medium surrounding the NWs is valid for low concentrations of chiral molecules deposited in the solution which evaporates, leaving the molecules in the NW vicinity. In future work, the experimental proof of principle will be done for chiral molecules that have intrinsic circular dichroism at the modal resonances of a chosen Si nanowire array.

## 5. Conclusions

In this work, we have proposed a path to enhanced near-field optical chirality by means of symmetric Si NW arrays, which support leaky waveguide modes that enhance the near-field optical chirality of CP excitation in the shorter wavelength part of the visible spectrum, which is of interest for many chiral molecules. The C enhancement can be optimized by choosing the wavelength range where enantiomers show CD, setting the radius, length and period of the NW array so that it gives resonances in that range, and optimizing the molecules position. Such an achiral approach does not suffer from the background chiral behavior present in intrinsically or extrinsically chiral plasmonic nanomaterials, and the absorption dissymmetry arises only for the near-field effects. Moreover, the use of conventional Si technology enables the functionalization of the NW surface which greatly enhances the overall chirality. We believe that this simple approach can lead to Si nanostructures-governed applications in enhanced enantioselectivity.
